# Editorial: The Digestive Tract of Cephalopods: At the Interface Between Physiology and Ecology

**DOI:** 10.3389/fphys.2018.01409

**Published:** 2018-10-09

**Authors:** Giovanna Ponte, Eduardo Almansa, Paul L. R. Andrews

**Affiliations:** ^1^Department of Biology and Evolution of Marine Organisms, Stazione Zoologica Anton Dohrn Naples, Italy; ^2^Association for Cephalopod Research ‘CephRes' Naples, Italy; ^3^Oceanographic Center of the Canary Islands, Spanish Institute of Oceanography Santa Cruz de Tenerife, Spain

**Keywords:** animal welfare, cephalopods, digestive system, early-life stages nutrition, physiology, paralarvae

The collection of papers included in this Research Topic represents the outcome of some of the activities of the COST Action FA1301, Cephs*In*Action. It emerged from a series of presentations delivered during a workshop in Cascais (Portugal; November 24th, 2015), and from the research activities carried out during Short Term Scientific Missions supported through the COST Action FA1301. The overall aim is to fill some lacunae in knowledge of the digestive tract of cephalopod molluscs. In contrast to other areas of cephalopod biology such as the central nervous system and behavior (e.g., Marini et al., 2017; Nakajima et al.; O'Brien et al.; Shigeno et al.) and the visual system (see Hanke and Osorio), relatively little research has been done on this topic during the last 30 years.

Cephalopods are active marine predators counting more than 800 species. Understanding the physiological adaptations of these fascinating and complex molluscs poses important challenges for several disciplines. Knowledge of the normal functioning (i.e., appetitive drive, signaling satiety, storage and coordinated oro-anal movement of ingested food and digesta, extra- and intra-cellular digestion, epithelial and intra-cellular transport, metabolism, and incorporation of nutrients in the tissues) of the digestive system has wide ranging implications for fisheries, aquaculture, and for the care and welfare of cephalopods in the laboratory and in public displays. Alterations in digestive tract functionality are also a sensitive indicator of gastrointestinal and systemic infections, disease, and external stressors in the broadest sense. Most of the available knowledge on the cephalopod “gut” and physiology of digestion is based on assumptions by analogy with the vertebrate digestive system.

This Research Topic includes 17 papers from more than 70 authors representing a contribution to the outcomes of COST FA1301. The papers present original data and/or reviews on: nutritional requirements and challenges offered by early-life stages, predatory behavior, anatomy and physiology of the cephalopod digestive system, and possible implications with animal care and welfare.

Among other species, the common octopus, *Octopus vulgaris*, is a prime species for cephalopod aquaculture but its potential is limited by poor survival during the paralarval stage. Limited knowledge of feeding habits and the digestive tract physiology are considered major barriers to progress and these areas are reflected by the nine paralarvae papers included here.

Nande et al. studied the predatory behavior and related movements of the digestive tract in 3-days post hatching (dph) *O. vulgaris* paralarvae hatched in the laboratory and fed on eighteen different types of wild caught prey. Capture and ingestion of decapod prey was less efficient (60%) than cladocerans or copepods (100%). Overall, paralarvae spent only ~5 min in contact with prey. The temporal sequence of digestive tract motility changes (e.g., crop and stomach filling, intestinal peristalsis) following food ingestion was quantified and pigmented food particles appeared in the digestive gland ~5 min after the crop had reached maximum volume.

Fernández-Gago et al. provide a 3D reconstruction of the digestive tract during the first 35 days of life, identifying four developmental periods (embryonic, early and late-post-hatching, and juvenile-adult), suggesting that the radula and digestive gland may take longer to mature than other regions. Despite the limitations of a morphological study, this paper provides background information against which the more functional studies can be considered.

A comparison by Estefanell et al. of wild caught with captive bred hatchlings highlights the potential utility of measurement of fatty acids such as *n*−3 highly unsaturated fatty acids from neutral and polar lipids in elucidating the nutritional requirements of *O. vulgaris* paralarvae. Lourenço et al. analyzed the lipid class content and fatty acid profiles of wild paralarvae and their potential prey and proposed that monounsaturated fatty acids (particularly C18:1n7) and the DHA:EPA ratio are trophic markers of the diet of paralarvae. The search for nutritional imbalance biomarkers is explored further by Morales et al. who measured changes in anaerobic and aerobic metabolism, fatty acid oxidation, and gluconeogenesis (from glycerol and amino acids) in *O. vulgaris* paralarvae during an extended part of this life-stage. Authors' findings suggest that phospholipid and n-3 HUFA-enriched *Artemia* reduced mortality and increased paralarval growth, thus contributing to the understanding of the ontogeny of metabolic pathways, an essential requirement for optimizing the diet of paralarvae in culture. A similar dietary enrichment was used by García-Fernández et al. to investigate the epigenetic regulation by diet and age of octopus paralarvae. An age-related demethylation was observed during the first 28 days of life and was accelerated by dietary n-3 HUFA enrichment. A proteomic approach allowed authors to identify specificity in the diet (*Artemia* enriched with microalgae vs. crustacean zoeae), and allowed comparison of fed and food deprived paralarvae suggesting that arginine kinase, NAD+ specific isocitrate dehydrogenase and S-crystallin 3 may be useful as biomarkers of nutritional stress (Varó et al.). Metagenomics provided a different approach to assessing diet in wild paralarvae, by analysis of DNA from the dissected digestive gland (Olmos-Pérez et al.) to identify Molecular Taxonomic Units recognizing decapods, copepods, euphausiids, amphipods, echinoderms, molluscs, and hydroids as part of the natural diet. Some paralarvae showed a preference for cladocerans (see also Nande et al.) and ophiuroids and overall seasonal variability was shown in the presence of copepods and ophiuroids in the diet.

Roura et al. investigated the paralarval microflora (microbiome). Both wild caught paralarvae and those newly hatched in captivity had similar microbial communities which the authors termed the “Core Gut Microflora,” the presence of which they considered indicative of healthy *O. vulgaris* paralarvae. A finding of particular relevance to aquaculture was that after 5 dph, in comparison to newly hatched paralarvae the number of bacterial species was reduced by ~50% with two families (Mycoplasmataceae and Vibrionacea) dominating. The importance of the microbial diversity provided by zooplankton in the wild in contrast to the typically used *Artemia* diet in captivity is discussed by the Authors.

A short overview of cephalopod predatory habits is also included in this Research Topic (Villanueva et al.) including an account of the relative roles of photo-, mechano-, and chemo-reception in the detection of diverse prey types in relation to living habits (e.g., photic zone, primarily vision; deep sea, primarily mechanoreception). A variety of hunting strategies (ambushing, luring, pursuit, stalking, pouncing, cooperation, and scavenging) are employed by different cephalopod species and the authors make an interesting comparison with marine and terrestrial vertebrates. Attention is drawn to the neglected area of the ontogeny of predation by reference to the feeding behavior of both hatchlings and senescent cephalopods.

A contribution to the anatomy and physiology of the digestive system of cephalopods is given by five papers. Ponte and Modica review the largely overlooked topic of the evolution of the salivary glands in molluscs by comparing gastropod and cephalopod molluscs, including a detailed tabulated comparison of the saliva constituents, and the relative role of the secretions from the submandibular gland, the anterior and posterior salivary glands in prey immobilization (by neurotoxins such as cephalotoxin and tetrodotoxin) and digestion (by enzymes). The subsequent steps in digestion are described in detail in *Octopus maya* and *Octopus mimus* by Gallardo et al. by highlighting novel data on the temporal pattern of absorption and assimilation, and providing preliminary evidence that lipid mobilization is dependent upon habitat water temperature.

The digestive gland in cephalopods is the main organ of metabolism and is analogous to the vertebrate liver. It secretes a range of digestive enzymes into the lumen of the digestive tract, receives digested nutrients from the caecum which it assimilates and subsequently transfers to the haemolymph (glucose and lipids). The digestive gland (DG) is also the main site of detoxification and storage of ingested marine pollutants as reviewed by Rodrigo and Costa. High concentrations of both essential (e.g., Cu and Zn) and non-essential (e.g., Ag, Cd, and Pb) metals with metal homeostasis involving *spherulae* formation, chelation and metallothionins characterize the DG. The authors also discuss the involvement of the DG in the storage and metabolism of organic toxicants including amnesic shellfish toxins (e.g., domoic acid), polycyclic aromatic hydrocarbons and polychlorinated biphenyls, and comparisons made with the mechanisms operating in the vertebrate liver including biotransformation, conjugation and elimination with a focus on the cytochrome P450 system.

Understanding the metabolic adaptations of cephalopods to environmental changes is of growing importance because of predicting the effects of climate change and the consequences of coastal eutrophication and assessing the impact of intensive aquaculture. Capaz et al. reported that exposure of adult cuttlefish to sea water with a 50% decreased oxygen for 1 h markedly increased breathing frequency (85%) and reduced oxygen consumption (37%), but there was only a small increase in mantle muscle octopine levels indicative of anaerobic metabolism. Complementary *in vitro* studies of protein turnover and Na^+^/K^+^ATPase activity (responsible for ionic gradient maintenance) enabled the authors to hypothesize that the reduced oxygen consumption in hypoxic animals was primarily due to reduced protein synthesis and Na^+^/K^+^ATPase activity.

In *O. vulgaris* Baldascino et al. utilized RT-PCR to reveal the neurochemical complexity of the gastric ganglion with evidence for putative peptide and non-peptide neurotransmitters (e.g., cephalotocin, FMRFamide, and 5-hydroxytryptamine) and/or their receptors (e.g., cholecystokinin_A, B_ and orexin_2_). A comparison of gene expression in the gastric ganglion of animals with relatively high or low levels of infection with the common digestive tract parasite *Aggregata octopiana* showed differential gene expression (e.g., increased NFκB, toll-like 3 receptor and decrease superoxide dismutase and glutathione peroxidase).

The regulation in European Union states of scientific research utilizing cephalopods has necessitated the development of guidelines for their care and welfare in the laboratory (Fiorito et al., 2015). Monitoring the functionality of the digestive tract is an important aspect of objective assessment of the overall welfare of captive cephalopods in general and particularly following an experimental procedure. A contribution to care and welfare as identified through the functioning of the digestive system is provided by two papers.

Ponte et al. survey non-invasive methodology for monitoring the physiology of the digestive tract in cephalopods *in vivo*. Measuring the predatory responses, measuring food intake or body weight by non-invasive approaches or measurement of oro-anal transit time (imaging and markers) are challenges that can possibly be overcome by using tools such as ultrasound to monitor movements of the digestive tract or fecal analysis as a “reporter” of digestive tract function.

A wide-ranging overview of the relevance of understanding digestive tract functionality to the welfare of cephalopods in the laboratory and aquaculture is given by Sykes et al. Authors discuss the challenges of feeding cephalopods in captivity and particularly issues around: live food and prepared diets, feeding frequency and quantity, the impact of a range of experimental interventions (e.g., surgery) on the digestive tract, and a discussion of the impact of food deprivation on the overall health and welfare of the animal.

## Author contributions

All authors have made a substantial, direct and intellectual contribution to the editorial.

### Conflict of interest statement

The authors of this editorial are co-authors of one or more of the publications discussed in this Editorial. The authors declare that the research was conducted in the absence of any commercial or financial relationships that could be construed as a potential conflict of interest.
